# Plasma metabolic profiling analysis of Cortex Periplocae-induced cardiotoxicity based on UPLC/Q-TOF-MS

**DOI:** 10.1039/c7ra12247k

**Published:** 2018-01-29

**Authors:** Yubo Li, Chuanxin Liu, Jun Du, Xue Sheng, Yanjun Zhang

**Affiliations:** Tianjin State Key Laboratory of Modern Chinese Medicine, School of Traditional Chinese Materia Medica, Tianjin University of Traditional Chinese Medicine 312 Anshan West Road Tianjin 300193 China tianjin_tcm001@sina.com +86-22-59596221 +86-22-59596221; Tianjin State Key Laboratory of Modern Chinese Medicine, Tianjin University of Traditional Chinese Medicine 312 Anshan West Road Tianjin 300193 China

## Abstract

Cortex Periplocae is a well-known form of traditional medicine with its unique cardiotonic action, anti-tumor activity and immune regulation effect. However, improper use of Cortex Periplocae often leads to cardiac toxicity, which in the most severe cases can even be life-threatening. Biochemical tests and histopathological examinations are primary methods for clinical trials. However, such approaches are time-consuming, lack specificity and have low sensitivity, which can easily lead to negative results in studies. Therefore, a more scientific and systematic evaluation of Cortex Periplocae cardiotoxicity is particularly important. In this study, we established a method that combines metabonomics with trend analysis of a gavage concentration series to find cardiac toxicity biomarkers of Cortex Periplocae. We created rat cardiotoxicity models, in which the toxicity was caused by Cortex Periplocae. We collected data from rat plasma samples based on metabonomics using ultra-performance liquid chromatography quadrupole time-of-flight mass spectrometry (UPLC/Q-TOF-MS). Multiple statistical analyses, such as principal component analysis (PCA) and partial least squares-discriminant analysis (PLS-DA), were used to examine metabolite profile changes in plasma samples to screen potential cardiotoxicity biomarkers and metabolic pathways. Compared with the control group, after 7 days administration, the pathological sections showed cardiac toxicity. Moreover, some metabolites in the body changed significantly. Receiver operating characteristic curve (ROC) analysis showed that there are 11 metabolites related with cardiac toxicity, which play a role in “phenylalanine, tyrosine and tryptophan biosynthesis”; “phenylalanine metabolism”; “valine, leucine and isoleucine biosynthesis”; “glycerophospholipid metabolism” as well as “pantothenate and CoA biosynthesis”. These metabolites can better explain the cardiotoxicity mechanism of Cortex Periplocae and provide a scientific and systematic method to evaluate the cardiotoxicity of Cortex Periplocae.

## Abstract 2

The experimental design flow for screening the cardiotoxicity biomarkers induced by Cortex Periplocae.

## Graphical abstract

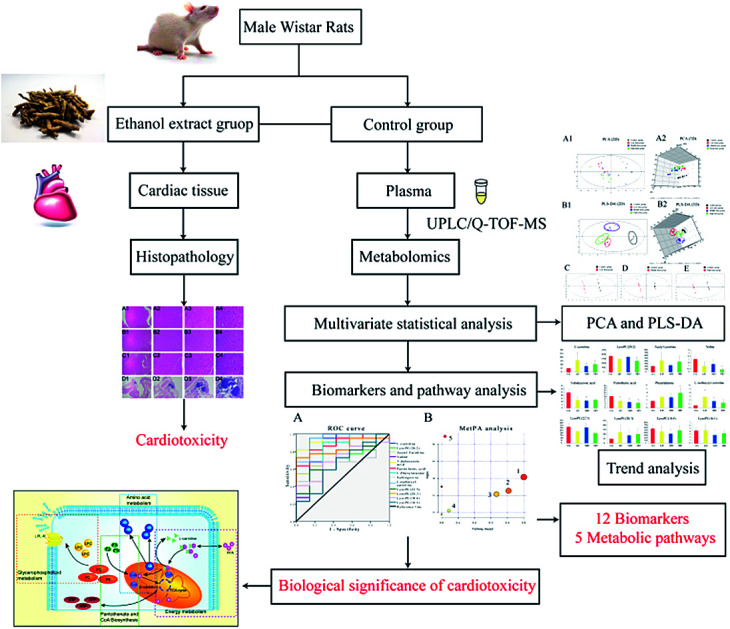

## Introduction

Cortex Periplocae, the root of Periploca sepium Bge, is a famous and commonly used traditional Chinese medicine (TCM) with a long history in China. It is often used for the clinical treatment of rheumatoid arthritis and chronic congestive heart failure. The 2015 edition of the Pharmacopoeia of People's Republic of China recorded Cortex Periplocae as toxic.^1^ The main toxic components are Periplocin and its aglucon, which are classified as cardiac glycosides.^2–4^ These toxic substances, used improperly, can easily trigger cardiotoxicity. Considering the wide application of Cortex Periplocae in clinics, it is necessary to develop an accurate and reliable method to assess the safety and toxicity of Cortex Periplocae. Biochemical and histological examinations are the main methods for conventional drug safety evaluation. However, they often lack sensitivity and specificity and thus, it is difficult to assess the toxicity of Cortex Periplocae, as well as other TCMs, ethnic herbs and natural drugs due to the complexity of the components.^5,6^

Metabonomics, as a novel “-omics” technology in the post gene era, is defined as “the quantitative measurement of the dynamic multiparametric metabolic response of living systems to pathophysiological stimuli or genetic modification”.^7^ The comprehensive metabonomic approach is in line with the dynamic and holistic concept of TCM. Application of metabonomics in the evaluation of toxicity and to validate the effect of TCM has been appreciated and performed, which provides rapid, highly sensitive and high throughput analysis. Therefore, it can better reveal the different endogenous substances in the body before and after administration in physiology and pathology. Simultaneously, it can provide some guidance for the research of the generation and development of the disease and the mechanism of drug toxicity.^8–13^

Nuclear magnetic resonance spectroscopy (NMR), gas chromatography combined with mass spectrometry (GC-MS), liquid chromatography combined with mass spectrometry (LC-MS), and capillary electrophoresis combined with mass spectrometry (CE-MS) are the most widely used analytical platforms in metabonomics studies. Among these, ultra-performance liquid chromatography (UPLC) coupled with MS leads to a considerable decrease in the analysis time and increase in sensitivity; it has been considered to have a brighter future in the research of metabonomics.^14,15^ A targeted method based on ultra-performance liquid chromatography/triple quadrupole-mass spectrometry (UPLC/QqQ-MS/MS) and a non-targeted method, based on ultra-performance liquid chromatography/quadrupole-time of flight-mass spectrometry (UPLC/Q-TOF-MS) are two major branches of the same field of metabonomics. Multiple reaction monitoring (MRM) in triple quadrupole mass spectrometry is widely used for targeted metabonomics or the low abundance analysis of small molecules *in vivo*. This approach has already established several clear superiorities, such as high sensitivity, wider linearity, better repeatability and quantitative accuracy. For example, Tie *et al.*^16^ used the UPLC-MRM platform to develop an innovative derivatization method that can improve the detection sensitivity of endogenous fatty aldehydes by a thousand times with accurate quantification. However, the targeted method is handicapped by its near-total dependency on standard products to obtain the information of ion pairs. Because of its inherent drawbacks, this method is under limited development. In contrast, the non-targeted method based on UPLC/Q-TOF-MS is a commonly employed approach in metabonomics study, which is able to detect as many metabolites as possible without previous knowledge of the sample components.^17,18^ For example, Fan *et al.*^19^conducted a clinical non-targeted metabonomics study based on LC/Q-TOF-MS using a large-scale sample of 2324 metabonomics cases. From this sample, The Atlas of Clinical Classification of Coronary Heart Disease was drawn for the first time. However, the primary disadvantage of this method is that its sensitivity and reproducibility are poor. In general, each method has a distinct approach for monitoring and obtaining data, and each of them has distinct advantages and disadvantages. At present, non-targeted metabonomics is still a mainstream and extensive application of the study of metabonomics. Examples include human hepatocellular carcinoma,^20–22^ acute and chronic liver failure,^23^ lung cancer,^24,25^ acute kidney injury^26^ and chronic renal failure.^27,28^ In addition, by combining the advantages of the two methods, a pseudotargeted method was proposed to analyze all detectable components in selected ion monitoring (SIM) mode^29^ by GC-MS or MRM mode by LC-MS.^30^

Up to now, metabonomics still has a vital catalytic role in evaluating the toxicity of TCM. Qualitative analysis and quantitative detection of metabonome can evaluate metabolic responses to reveal the differences in metabolic levels between control and TCM-administration groups. A package of successful metabonomic applications in important TCM fields could assess toxicity at the early stages and find out the biological markers at the metabolic level to clarify the possible toxic mechanisms *in vivo*.^31–34^ Numerous studies have employed metabonomics to investigate biomarkers for the toxicity of TCM, including cardiotoxicity (Aconiti Radix,^32,35^ and Radix Aconiti Preparata^36^), hepatotoxicity (Cimicifugae Rhizoma^37^ and Psoraleae Fructus^38^), nephrotoxicity (Tripterygium Wilfordii Hook. F.^39^) and neurotoxicity (Acanthopanax senticosus^40^). In addition, simplex components of TCM (aristolochic acid^41,42^ and triptolide^43^) and compound prescription (Niuhuang Jiedu Tablet^44,45^) were also performed by the metabonomics approach.

In light of this, we performed a systematic metabonomic approach to evaluate the metabolic response of Cortex Periplocae to find out the key biomarkers to assess Cortex Periplocae induced cardiac toxicity. UPLC/Q-TOF-MS was used for the metabolic profiling analysis of plasma samples of rats treated with different concentrations of Cortex Periplocae to find the cardiotoxicity biomarkers. A histopathology examination assay was also performed. It showed that the Cortex Periplocae-administration group damaged the cardiac tissue to some extent. In total, 11 cardiotoxicity biomarkers have already been found and the sensitivity and specificity of these biomarkers were evaluated *via* the receiver operating characteristic curve (ROC curve). Combined with the biological significance of biomarkers, the possible mechanism of Cortex Periplocae-induced cardiotoxicity at metabolic level can be explained. Our results provide a reference for the understanding of Corex Periplocae-induced cardiac toxicity mechanisms and also promote the development and application of metabonomics in the field of TCM toxicity evaluation.

## Experimental

### Reagents and materials

High pressure liquid chromatography (HPLC)-grade acetonitrile and formic acid were purchased from Oceanpak (Gothenburg, Sweden) and ROE (USA), respectively. Distilled water was obtained from Wahaha (Hangzhou, China). Cortex Periplocae was purchased from Hebei Jin Mu Pharmaceutical Group Co., Ltd. and had been identified by pharmacognosy experts.

### Extraction of TCM

In this study, we crushed Cortex Periplocae herbs; 67.5 g, 135 g, and 202.5 g of the crushed herb were extracted twice with 10 and 8 times the volume of 70% ethanol, in sequence. Then, the extracting solutions were refluxed for 2 h and then filtered, combined, and concentrated to 0.3 g mL^−1^, 0.6 g mL^−1^ and 0.9 g mL^−1^.

### Animal treatment

The experimental animals were purchased from Vital River Laboratory Animal Technology Co., Ltd (Beijing), with the license number “SCXK (Jing) 2012-0001”. We performed animal experiments at the Institute of Radiation Medicine, Chinese Academy of Medical Sciences (Tianjin, China). In total, 36 male Wistar rats weighing 200 ± 20 g were raised in an SPF-level lab, which were randomly divided into control group (only treated with distilled water), low dose group (only treated with ethanol extract of Cortex Periplocae (3 g kg^−1^)), middle dose group (only treated with ethanol extract of Cortex Periplocae (6 g kg^−1^)) and high dose group (only treated with ethanol extract of Cortex Periplocae (9 g kg^−1^)). The rats were housed under the following conditions: 7 days, ambient temperature of 23 ± 2 °C and humidity of 35 ± 5%. The groups, doses, administration modes and sampling times are listed in Table 1. This study was approved by the Animal Ethics Committee of Tianjin University of Traditional Chinese Medicine under permit number TCM-2012-078F01. All experimental procedures were conducted in accordance with Chinese national legislation and local guidelines.

**Table 1:** Dose, mode of administration and sampling time in the process of screening and validating biomarkers

Grouping	Drug	Num	Dose	Mode	Time
Control group	Distilled water	9	2.5 ml	ai.g., successive administration	7 days
Low dose group	Cortex Periplocae	9	3 g kg^−1^ d^−1^	ai.g., successive administration	7 days
Middle dose group	Cortex Periplocae	9	6 g kg^−1^ d^−1^	ai.g., successive administration	7 days
High dose group	Cortex Periplocae	9	9 g kg^−1^ d^−1^	ai.g., successive administration	7 days

a Intragastric administration.

### Sample collection and preparation

After treatment with Cortex Periplocae, the blood samples were collected 7 days after administration. Before sample collection, all animals were fasted for 12 h, but water was allowed, to avoid the effects of food on the final results. Abdominal aortic blood samples were collected after all animals were anesthetized with chloral hydrate. Following this, all animals were sacrificed, and the hearts were immediately removed and fixed in 10% formalin solution. The blood samples were centrifuged at 760 × *g* for 15 min. The obtained supernatant was centrifuged at 1040 × *g* for 8 min. Then, we extracted the supernatant. The plasma samples were stored at −80 °C before the metabonomic analysis. Plasma samples removed from the −80 °C refrigerator were thawed at room temperature. The plasma of 9 centrifuge tubes of the control group (200 μL per tube) were mixed together to prepare quality control (QC) samples, which contained all plasma information. We used 300 μL acetonitrile in 100 μL plasma or QC sample for the protein precipitation. The resultant mixture was ultrasonicated in cold water for 10 min, vortexed for 1 min and then centrifuged at 14 360 × *g* for 15 min at 4 °C. The supernatants were used for the metabonomic analysis. The QC samples were utilized to optimize and supervise the UPLC-MS analysis process. The pathological features of the tissues were examined by haematoxylin and eosin (H&E) staining. Histopathological changes were identified under a light microscope at 40×, 100×, 200× and 400× magnifications.

### Chromatographic and mass spectrometric conditions

HPLC analysis was performed using a Waters UPLC/Q-TOF-MS system (Waters, USA). Plasma samples (5 μL) were injected into an ACQUITY UPLC HSS C18 column (2.1 × 100 mm, 1.7 μm, Waters). The column temperature was set to 40 °C, and the flow rate was set to 0.3 mL min^−1^. The UPLC separation system includes a binary solvent system with mobile phase A (0.1% formic acid in water) and mobile phase B (0.1% formic acid in acetonitrile). The gradient profiles for the plasma samples were as follows: 99% A; followed by 0–0.5 min, A: 99–99%; 0.5–2 min, A: 99–50%; 2–9 min, A: 50–1%; 9–10 min, A: 1–1%; 10–10.5 min, A: 1–99%; 10.5–12 min, A: 99–99%. Q-TOF-MS was equipped with electrospray ionisation in positive mode. The MS parameters were as follows: drying gas temperature, 325 °C; drying gas flow, 10 mL min^−1^; desolvation gas flow, 600 L h^−1^; capillary voltage, 2.1 kV; fragmentor voltage, 6 kV; collision energy, 20–30 kV; nebulizer pressure, 350 psi; and evaporative gas and auxiliary gas, high purity nitrogen; reference ions ([M + H]^+^ = 556.2771, [M − H]^−^ = 554.2615) were used to ensure accuracy during spectral acquisition. The range of data acquisition was 50–1000 Da. All samples were randomly injected. The samples were singled out from each group and mixed together to make quality control (QC) samples. Containing all plasma information, the QC samples were used to optimize and supervise the analysis process.^46,47^ QC samples were injected alternatively (every 10 samples) to test the stability of the samples and the system during acquisition.

### Data processing

In this experiment, in order to validate the LC/MS analysis, the precision, reproducibility and stability of the specimens were determined according to the QC samples. The retention time and the relative content of the metabolites differed within the spectrum. Twenty of these samples were randomly selected to evaluate the relative standard deviation (RSD) of precision and reproducibility. The raw data of the control and model groups were collected with MarkerLynx Version 4.1 (Waters Corp., Manchester, USA) based on the UPLC/Q-TOF-MS. The data were exported after normalization. Then, the data were imported into simca-p11.5 software (Sweden Umetrics Company) for multivariate statistical analysis after 80% revision (Excel format). Principal component analysis (PCA) and partial least squares discriminant analysis (PLS-DA) was used to establish the model. Based on this model, the material with a VIP (Variable Importance Plot) greater than 1 is selected as a potential biomarker. The statistical significance was *P* <0.05 (student's *t*-test). In order to obtain an accurate molecular weight, we used the HMDB database to retrieve *m*/*z* values (http://www.hmdb.ca). Through screening endogenous substances, biomarkers were further identified. The final determination of biomarkers was carried out using two-stage mass spectrometry. The heat map was generated using cluster software based on the relative content of each metabolite. The ROC curves of cardiotoxicity biomarkers based on the Cortex Periplocae administered group were determined using the binary logistic regression model in SPSS 17.0 (SPSS, USA).

## Results and discussion

### Method validation

In this study, QC samples were prepared in parallel and injected to evaluate the repeatability of the method. Moreover, a QC sample was injected to ensure that the samples and the instrument were stable within 24 h. In total, 20 chromatographic peaks were chosen randomly and the RSD of peak areas and the retention time of these peaks for instrument precision, method repeatability and sample stability were calculated and assessed. The results of the methodology are summarized in Table 2; the instrument precision, method repeatability and sample stability are all in line with the requirements of metabonomics (RSD < 15%).

**Table 2:** The results of the experimental methodology

Experiment name	RSD (peak area)	RSD (retention time)
Instrument precision	<10%	<1.0%
Method repeatability	<10%	<1.0%
Sample stability	<15%	<1.0%

### Histopathological evaluation

**Fig. 1:**
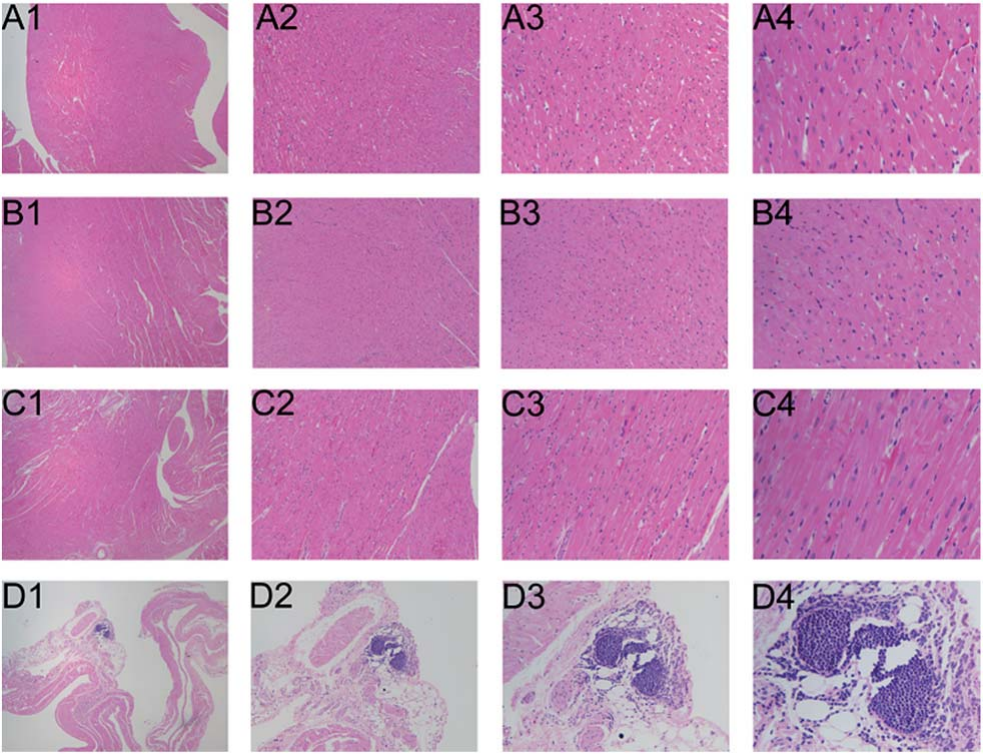
Low dose group (B1–B4), middle dose group (C1–C4) and high dose group (D1–D4) post-Cortex Periplocae administration for the heart tissue were assessed by histopathology compared with control group administration (A1–A4). Histopathological changes were identified under a light microscope at 40× (A1–D1), 100× (A2–D2), 200× (A3–D3) and 400× (A4–D4) magnification.

The histopathological results demonstrated that as compared with to control group, the middle dose and high dose of Cortex Periplocae administration groups exhibited heart tissue injury, which can be manifested as eosinophilic degeneration of part of the muscle fibers and cell invasion. The cardiac tissue of the low dose group did not exhibit damage (Fig. 1).

### Metabolic profiling analysis

**Fig. 2:**
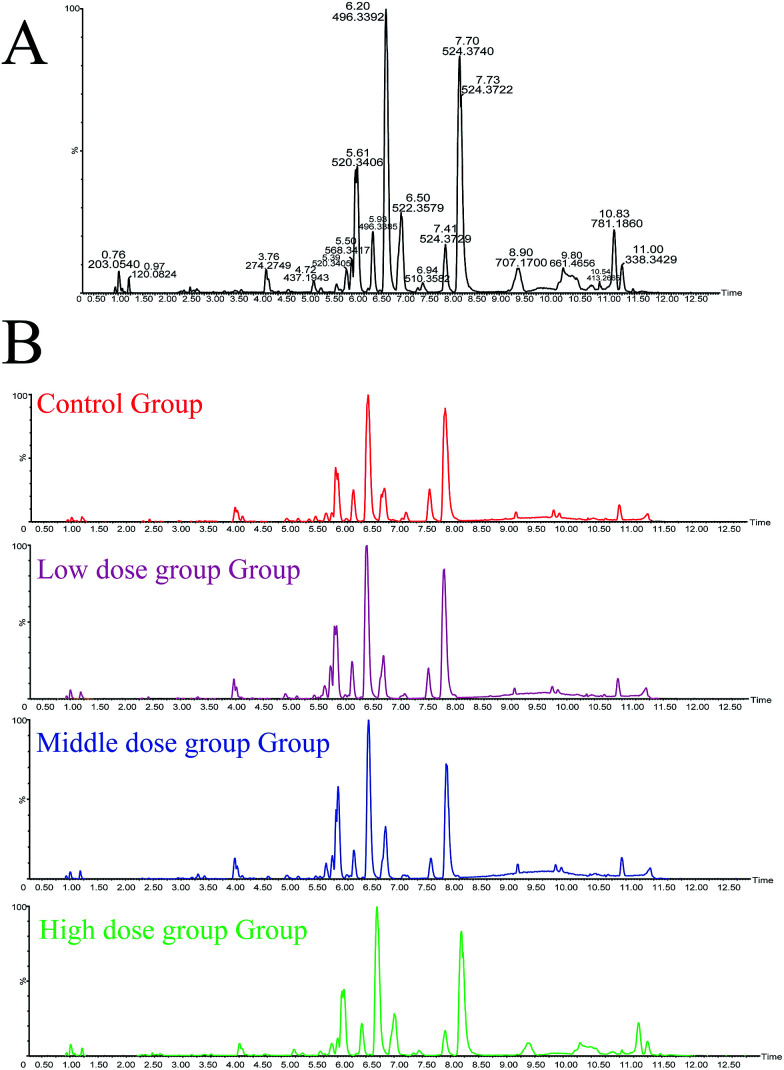
(A) The base peak intensity (BPI) chromatogram of plasma in the QC sample in positive mode was obtained based on the UPLC/Q-TOF-MS platform. (B) The base peak intensity (BPI) chromatogram of plasma in the control group and Cortex Periplocae administered group in positive mode were obtained based on the UPLC/Q-TOF-MS platform.

**Fig. 3:**
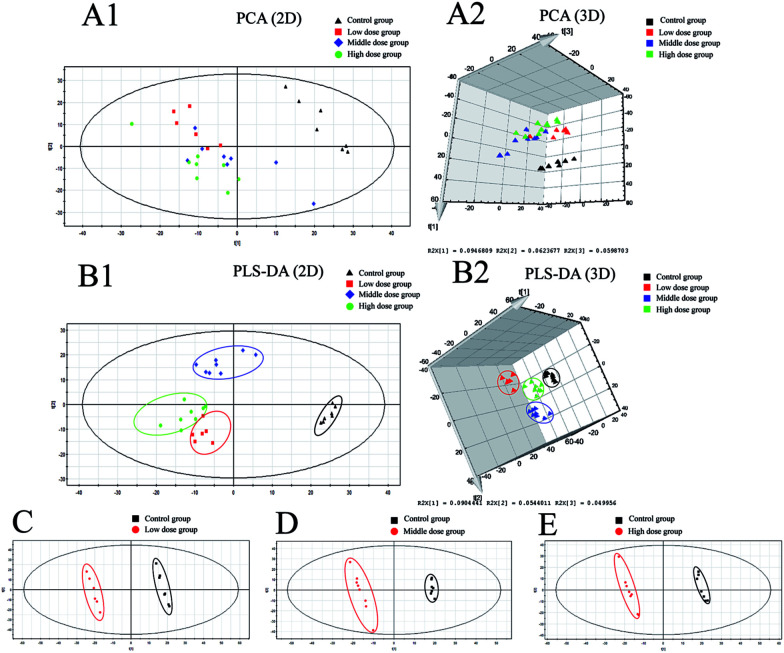
Result of multivariate statistical analysis. (A1) PCA 2D scores plots of low dose groups, middle dose groups and high dose groups of alcohol extract of Cortex Periplocae compared with control groups (*R*^2^*X* = 0.727 *Q*^2^ = 0.764); (A2) PCA 3D scores plots of low dose groups, middle dose groups and high dose groups of alcohol extract of Cortex Periplocae compared with control groups; (B1) PLS-DA 2D scores plots of low dose groups, middle dose groups and high dose groups of alcohol extract of Cortex Periplocae compared with control groups (*R*^2^*X* = 0.284 *R*^2^*Y* = 0.989 *Q*^2^ = 0.746); (B2) PLS-DA 3D scores plots of low dose groups, middle dose groups and high dose groups of alcohol extract of Cortex Periplocae compared with control groups. (C–E) PLS-DA scores plots of low dose groups (*R*^2^*X* = 0.42, *R*^2^*Y* = 1, *Q*^2^ = 0.956), middle dose groups (*R*^2^*X* = 0.23, *R*^2^*Y* = 0.998, *Q*^2^ = 0.749) and high dose groups (*R*^2^*X* = 0.233, *R*^2^*Y* = 0.999, *Q*^2^ = 0.785) of alcohol extract of Cortex Periplocae compared with control groups, respectively.

Details of ion chromatography and mass spectrometry are shown in Fig. 2. We obtained the PCA (*R*^2^*X* = 0.727, *Q*^2^ = 0.764) and PLS-DA (*R*^2^*X* = 0.284 *R*^2^*Y*, = 0.989, *Q*^2^ = 0.746) score plots using multivariate statistical analysis (Fig. 3). Partial stray samples were removed according to PCA. As shown in the scatter diagram of PCA, after administration of Cortex Periplocae, there were some changes in the rat plasma. This indicated that the normal level of metabolism of rats was affected by Cortex Periplocae. Compared with unsupervised PCA, supervised PLS-DA can be used to obtain specific variables that cause differences among groups. A scatter plot of the PLS-DA model is also shown in Fig. 3. Observation by PLS-DA score profiles to groups of Cortex Periplocae doses are distributed in different regions. This showed that there were significant differences in the metabolic pattern of each drug group and the control group, and it had a strong predictive ability.

### Identification of biomarkers

The screening of biomarkers was determined according to the VIP parameters of PLS-DA analysis. In this study, VIP >1 was selected as the candidate biomarker. Possible endogenous plasma metabolites were sought in the HMDB database using the mass charge ratio of the compounds. The final identification of biomarkers were obtained according to two-stage mass spectrometry. One of the biomarkers (*t*_R_ = 5.8014 min, *m*/*z* = 494.3243) was used as an example to explain the process of identification of compounds. At first, we were using *m*/*z* in our search to obtain the molecular formula of compounds with the formula C_24_H_48_NO_7_P in the HMDB database. In addition, the mass spectra fragments of compounds at 476.3, 184.0, 125.0 and 104.1 *m*/*z* correspond to the loss of –H_2_O, –C_19_H_34_O_3_, –C_22_H_43_NO_3_ and –C_19_H_35_O_6_P, respectively. According to the fragment information, we eventually concluded that the compound was LysoPC(16:1). Identification results showed that when Cortex Periplocae was administered for 7 days, there were 12 different metabolites; the specific results are summarized in Table 3. In order to obtain accurate and high quality research results, in this study, VIP >1 (PLS-DA) and *P* <0.05 (student's *t*-test) represented potential biomarkers associated with cardiotoxicity.

**Table 3:** Twelve biomarkers related to Cortex Periplocae based on UPLC/Q-TOF-MS

No.	*t*_R_ (min)	Metabolites	Obsed *m*/*z*	Calcd *m*/*z*	ppm	Formula	Content variance^a^			MS/MS
							Low dose	Middle dose	High dose	
1	0.7676	l-Carnitine	162.1135	162.1130	3.08	C_7_H_15_NO_3_	↑**	↑**	↑**	162.1 [M + H]^+^
										103.0 [M + H − C_3_H_9_N]^+^
2	6.7406	LysoPC(20:2)	548.3717	548.3716	0.18	C_28_H_54_NO_7_P	↓	↓	↓**	548.3 [M + H]^+^
										184.0 [M + H − C_20_H_40_O_3_]^+^
										104.1[M + H − C_20_H_41_O_6_P]^+^
3	0.932	Acetyl carnitine	204.1243	204.1236	3.42	C_9_H_17_NO_4_	↑	↑	↑*	204.1 [M + H]^+^
										85.0 [M + H − N + (CH_3_)_3_ − CH_3_COOH]^+^
4	0.7688	Valine	118.0877	118.0868	7.62	C_5_H_11_NO_2_	↓*	↓	↓**	118.0 [M + H]^+^
										156.0 [M + K]^+^
										59.1 [M − C_3_H_8_N]^+^
5	8.6068	5-Dodecenoic acid	221.1531	221.1517	6.33	C_12_H_22_O_2_	↓**	↓**	↓**	221.2 [M + Na]^+^
										199.1 [M + H]^+^
										181.0 [M + H − H_2_O]^+^
6	0.9374	Pantothenic acid	220.1180	220.1185	−2.27	C_9_H_17_NO_5_	↓*	↓**	↓**	220.1 [M + H]^+^
										184.0 [M + H − 2H_2_O]^+^
7	2.0541	Phenylalanine	166.0874	166.0868	3.61	C_9_H_11_NO_2_	↑	↑	↑**	120.0 [M − C_3_H_9_]^+^
										91.0 [M − C_3_H_8_NO]^+^
										102.9 [M − C_2_H_8_NO]^+^
8	2.1636	l-Isobutyryl carnitine	232.1542	232.1549	−3.01	C_11_H_21_NO_4_	↑**	↑**	↑*	172.0 [M − C_3_H_9_N]^+^
										84.0 [M − C_7_H_18_NO_2_]^+^
9	6.741	LysoPC(22:5)	570.3521	570.3560	−6.83	C_30_H_52_NO_7_P	↓	↓	↓**	570.4 [M + H]^+^
										184.1 [M + H − C_21_H_41_NO_3_P]^+^
										125.0 [M + H − C_28_H_47_NO_3_]^+^
10	6.0194	LysoPC(20:3)	568.3379	568.3379	0	C_28_H_52_NO_7_P	↑*	↑*	↑*	568.3 [M + Na]^+^
										546.4 [M + H]^+^
										184.1 [M + H − C_18_H_37_NO_4_P]^+^
11	5.1854	LysoPC(18:4)	516.3060	516.3090	−5.81	C_26_H_46_NO_7_P	↓	↓**	↓	516.3 [M + H]^+^
										184.0 [M + H − C_21_H_32_O_3_]^+^
										104.1 [M + H − C_21_H_33_O_6_P]^+^
12	5.8014	LysoPC(16:1)	494.3243	494.3247	−0.81	C_24_H_48_NO_7_P	↑	↓*	↓	494.3 [M + H]^+^
										476.3 [M + H − H_2_O]^+^
										184.0 [M + H − C_19_H_34_O_3_]^+^
										125.0 [M + H − C_22_H_43_NO_3_]^+^
										104.1 [M + H − C_19_H_35_O_6_P]^+^

a ↑**, significantly increased compared with control group (*P* < 0.01); ↓**, significantly decreased compared with control group (*P* < 0.01); ↑*, significantly increased compared with control group (*P* < 0.05); ↓*, significantly decreased compared with control group (*P* < 0.05).

### Trend analysis of Cortex Periplocae induced-cardiac toxicity biomarkers

**Fig. 4:**
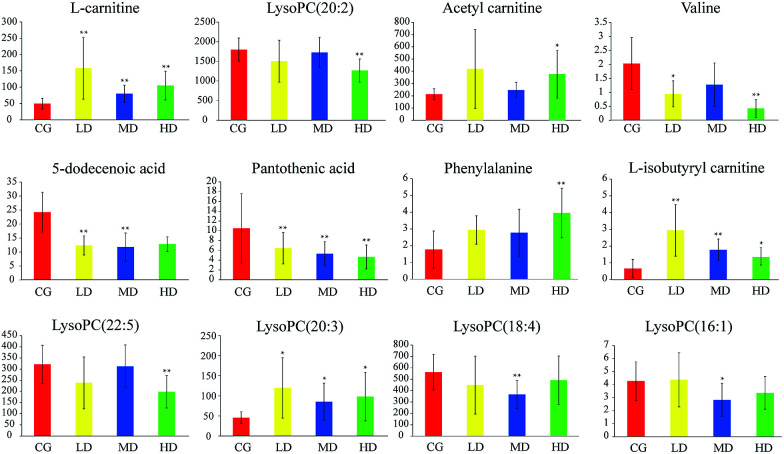
The tendency of 12 biomarkers'relative content (peak area intensity) changed with different concentrations of administration: CG (control group), LD (low dose group), MD (middle dose group), HD (high dose group). Significant difference from control: *p < 0.05, **p < 0.01.

With the dosage as the abscissa and the peak area of the biomarkers as the ordinates, histograms were created (see Fig. 4). The trends in biomarker variation were divided into two classes. Class(I) substances, such as l-carnitine and l-isobutyryl carnitine, had a tendency to increase. Among them, the most representative is phenylalanine, which has a significant correlation with dosage. Class(II) substances had a tendency to decrease. Pantothenic acid was the most pronounced in this process, which exhibited a downward trend successively with increasing dosage.

### Optimization by ROC curve

**Fig. 5:**
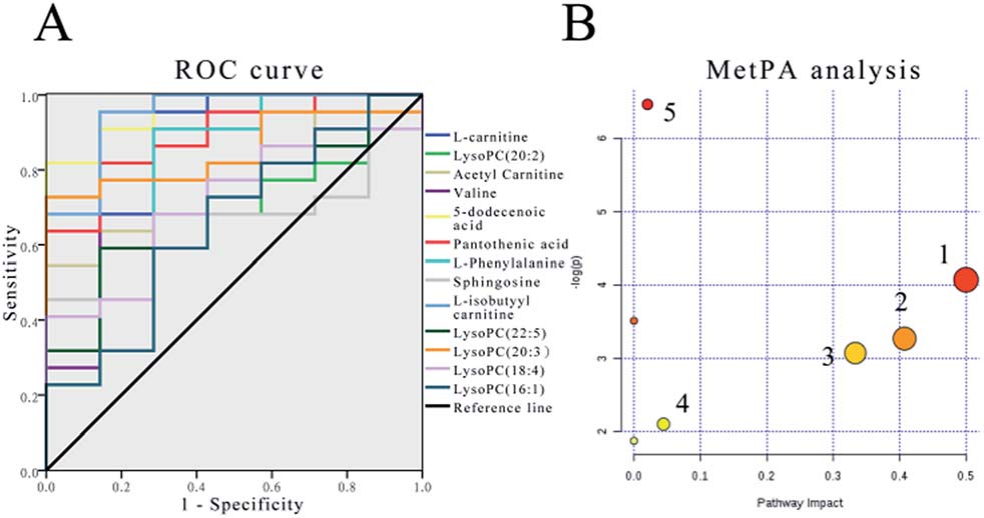
(A) The receiver operating characteristic (ROC) curve to assess the predictive ability of the biomarkers in alcohol extract of Cortex Periplocae. (B) Summary of pathway analysis: (1) phenylalanine, tyrosine and tryptophan biosynthesis; (2) phenylalanine metabolism; (3) valine, leucine and isoleucine biosynthesis; (4) glycerophospholipid metabolism; (5) pantothenate and CoA biosynthesis.

**Fig. 6:**
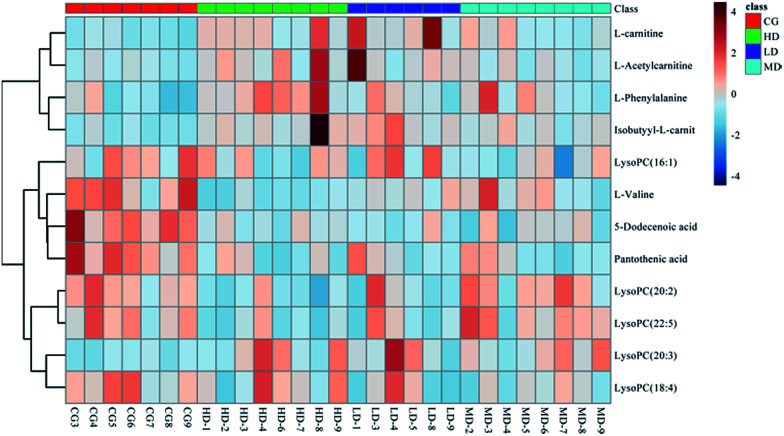
HCA/Heatmap hierarchical cluster analysis (HCA) of metabonomic data depicting the data structure of twelve biomarkers in low dose group (LD), middle dose group (MD), high dose group (HD) and control group (CG). Heatmap for metabolite changes color-scaled with correlation coefficients where warm color denotes an increase of metabolite levels whereas cold color (blue) indicates a decrease.

The sensitivity and specificity of the ROC curve are combined with graphical methods. As a comprehensive index to reflect the sensitivity and specificity of continuous variables, the diagnostic efficiency is usually evaluated by the area under the curve (AUC). If the AUC is greater than 0.7, the biomarkers are relatively exclusive and can be employed as diagnostic biomarkers.^48,49^ In this study, the ROC curve was used to investigate the diagnostic ability of these cardiac biomarkers for cardiac toxicity. The results are shown in Fig. 5(A) and Table 4. The results showed that under the condition of 95% confidence interval of ROC curve, the AUC of 12 markers was between 0.656 and 1.000 (Table 4). In addition, the 11 cardiac biomarkers had high sensitivity and specificity in evaluating the cardiac toxicity (AUC > 0.7). In order to visually analyze the metabolite changes *in vivo*, we used hierarchical cluster analysis, namely, Heatmap to analyze the biomarkers. The Heatmap visually showed 12 distinct diagnostic biomarkers in the low dose group, middle dose group, high dose group and control group. Fig. 6 shows the variable information as the ordinate and the sample information as the abscissa; the depth of the color represents the size of the variable. The closer the bifurcation of the variable information in the vertical axis, the higher is the similarity between these substances; they were probably derived from metabolites of the same substance. As shown in the figure, the levels of the 12 diagnostic biomarkers in the control group were significantly different from those in the low dose group, middle dose group and the high dose group.

**Table 4:** ROC curve analysis of the biomarkers

Biomarkers	AUCs	Std. error^a^	95% confidence interval
l-carnitine	0.903	0.064	0.000–1.000
LysoPC(20:2)	0.714	0.095	0.528–0.901
Acetyl carnitine	0.779	0.087	0.609–0.950
Valine	0.838	0.099	0.000–1.000
5-Dodecenoic acid	0.961	0.034	0.000–1.000
Pantothenic acid	0.890	0.063	0.741–1.000
l-isobutyyl carnitine	0.818	0.101	0.597–1.000
LysoPC(22:5)	0.948	0.047	0.000–1.000
LysoPC(20:3)	0.701	0.104	0.498–0.905
LysoPC(18:4)	0.851	0.071	0.712–0.989
LysoPC(16:1)	0.714	0.101	0.516–0.913
l-isobutyyl carnitine	0.656	0.120	0.421–0.891

a Under the nonparametric assumption.

### Metabolomic pathway analysis

The MetPA database was used for metabolic pathway analysis of potential biomarkers. The results are shown in Fig. 5(B). In this study, the metabolic pathways with an impact greater than 0.02 were considered to be associated with the Cortex Periplocae-induced cardiotoxicity. The impact represents the close link between metabolic pathways and toxicity; the specific information is illustrated in Table 5. The analysis results showed that five kinds of metabolic pathways were screened by MetPA, including “phenylalanine, tyrosine and tryptophan biosynthesis”, “phenylalanine metabolism”, “valine, leucine and isoleucine biosynthesis”, “glycerophospholipid metabolism” as well as “pantothenate and CoA biosynthesis”.

**Table 5:** MetPA analysis of 12 biomarkers

No.	Pathway name	*p*	−log(*p*)	Impact
1	Phenylalanine, tyrosine and tryptophan biosynthesis	0.017	4.073	0.500
2	Phenylalanine metabolism	0.038	3.271	0.407
3	Valine, leucine and isoleucine biosynthesis	0.046	3.074	0.333
4	Glycerophospholipid metabolism	0.122	2.104	0.044
5	Pantothenate and CoA biosynthesis	0.002	6.460	0.020

### Biological significance of biomarkers for cardiotoxicity

**Fig. 7:**
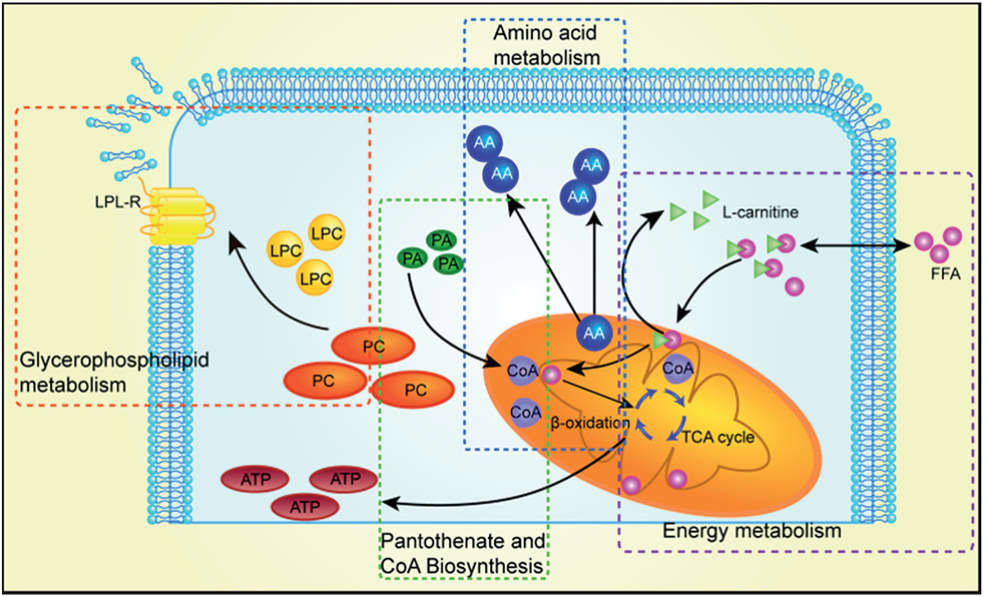
Different metabolites and corresponding pathways in Cortex Periplocae-treated rats.

The lysophospholipid receptor (LPL-Rs) is a member of the G protein-coupled receptor family and acts as a messenger during life activities.^50^ LPC substances, that is, lysophosphatidylcholine, belong to the PC class of phospholipids. We infer that certain factors may activate phospholipase A2, which leads to damage of the phospholipid membranes as well as LPC reduction, which increases heart damage.^51^ Carnitine substances have been considered to be reliable biomarkers to determine whether energy metabolism is abnormal.^52^ In our study, three carnitine species were found, l-carnitine, acetyl carnitine and isobutyryl-l-carnitine. Carnitine primarily transports activated fatty acids. It can be combined with long-chain saturated and unsaturated fatty acids, travel into the mitochondria through the mitochondrial membrane, and then provide energy by β-oxidation and tricarboxylic acid cycle reaction for the body's metabolic activity.^53^l-Carnitine plays an important role in the transport of fatty acids, and it may reduce mitochondrial dysfunction in cardiomyocytes.^54,55^ Cardiotoxicity can lead to increase in myocardial oxygen consumption, which may further aggravate the β-oxidation of fatty acids. Therefore, the levels of l-carnitine, acetyl carnitine and isobutyryl-l-carnitine increased significantly. The amino acid biosynthesis pathway is one of the basic metabolic pathways in the body, which can provide amino acids as raw materials for protein. Therefore, the balance of amino acid biosynthesis is of vital importance to the normal survival and growth of cells. In amino acid metabolism, phenylalanine is a necessary aromatic amino acid, which can produce ketone and carbohydrate substances and further involve in the tricarboxylic acid cycle to provide energy for the body. Therefore, when the heart is damaged, more phenylalanine is needed, providing energy during the repair process.^56^ Valine is a branched chain amino acid (BCAAs), which has an important role in the repair of cell membranes; hence, its content has been significantly reduced. Therefore, cardiotoxicity induced by Cortex Periplocae is multifaceted, which is a common result for multiple metabolic pathways. An illustration of the metabolic mechanism of Cortex Periplocae-induced cardiac toxicity is shown in Fig. 7.

## Conclusions

In this study, we have established a metabolic profiling analysis method based on UPLC/Q-TOF-MS to investigate Cortex Periplocae-induced cardiac toxicity in rats. Histopathological results demonstrated that compared with the control group, the middle dose and high dose of Cortex Periplocae administration groups exhibited heart tissue injury. Based on the above experiments, we found 12 biomarkers, which are significantly changed in the Cortex Periplocae group using multivariate statistical analysis compared with the control group. According to the optimizing function of the ROC curve, we screened 11 biomarkers, which contributed the most to the establishment of the model. In total, 11 highly-sensitive biomarkers can serve as an evaluation index of Cortex Periplocae-induced cardiac toxicity. Moreover, the AUC of one biomarker (l-isobutyyl carnitine) was less than 0.7 and should be neglected. Through metabolic pathway analysis, it could be concluded that the toxicity of Cortex Periplocae was attributed to the phenylalanine disruption, tyrosine and tryptophan biosynthesis, phenylalanine metabolism, valine, leucine and isoleucine biosynthesis, glycerophospholipid metabolism and pantothenate and CoA biosynthesis. By reflecting the changes of plasma metabolites, the method provides a more sensitive and systematic way to evaluate the cardiotoxicity of Cortex Periplocae, suggesting that the mechanism of cardiotoxicity might be linked to these pathways. In addition, the proposed method can also provide a reliable basis for the evaluation of drug safety and it also lays the foundation of research of the toxicity mechanisms of TCM.

## Conflicts of interest

The authors declare no competing financial interest.

## Supplementary Material
